# Co-cultures with stem cell-derived human sensory neurons reveal regulators of peripheral myelination

**DOI:** 10.1093/brain/awx012

**Published:** 2017-02-15

**Authors:** Alex J. Clark, Malte S. Kaller, Jorge Galino, Hugh J. Willison, Simon Rinaldi, David L. H. Bennett

**Affiliations:** 1 Nuffield Department of Clinical Neurosciences, West Wing, John Radcliffe Hospital, Oxford, UK; 2 Neuroimmunology Group, Institute of Infection, Immunity and Inflammation, College of Medical, Veterinary and Life Sciences, University of Glasgow, UK

**Keywords:** Schwann cell, myelin, Guillain-Barré syndrome, chronic inflammatory demyelinating neuropathy, stem cells

## Abstract

See Saporta and Shy (doi:10.1093/awx048) for a scientific commentary on this article.

Effective bidirectional signalling between axons and Schwann cells is essential for both the development and maintenance of peripheral nerve function. We have established conditions by which human induced pluripotent stem cell-derived sensory neurons can be cultured with rat Schwann cells, and have produced for the first time long-term and stable myelinating co-cultures with human neurons. These cultures contain the specialized domains formed by axonal interaction with myelinating Schwann cells, such as clustered voltage-gated sodium channels at the node of Ranvier and Shaker-type potassium channel (K_v_1.2) at the juxtaparanode. Expression of type III neuregulin-1 (TIIINRG1) in induced pluripotent stem cell-derived sensory neurons strongly enhances myelination, while conversely pharmacological blockade of the NRG1-ErbB pathway prevents myelination, providing direct evidence for the ability of this pathway to promote the myelination of human sensory axons. The β-secretase, BACE1 is a protease needed to generate active NRG1 from the full-length form. Due to the fact that it also cleaves amyloid precursor protein, BACE1 is a therapeutic target in Alzheimer’s disease, however, consistent with its role in NRG1 processing we find that BACE1 inhibition significantly impairs myelination in our co-culture system. In order to exploit co-cultures to address other clinically relevant problems, they were exposed to anti-disialosyl ganglioside antibodies, including those derived from a patient with a sensory predominant, inflammatory neuropathy with mixed axonal and demyelinating electrophysiology. The co-cultures reveal that both mouse and human disialosyl antibodies target the nodal axolemma, induce acute axonal degeneration in the presence of complement, and impair myelination. The human, neuropathy-associated IgM antibody is also shown to induce complement-independent demyelination. Myelinating co-cultures using human induced pluripotent stem cell-derived sensory neurons thus provide insights into the cellular and molecular specialization of axoglial signalling, how pharmacological agents may promote or impede such signalling and the pathogenic effects of ganglioside antibodies.

## Teaser

Clark *et al.* report that human sensory neurons derived from iPSCs can be myelinated by rat Schwann cells in culture. Such co-cultures develop features of mature myelinated fibres in a neuregulin-1 dependent manner. Antiganglioside antibodies associated with inflammatory peripheral neuropathies can both impede myelination and cause loss of established myelin.

## Introduction

There is a close anatomical and metabolic relationship between axons and Schwann cells such that the health of each cell type is intimately dependent on the other. Axoglial signalling is critical for myelination and can be disturbed in pathological states (Taveggia *et al.*, 2010). The use of primary rodent cells (dorsal root ganglion neurons and Schwann cells) in the generation of myelinating co-cultures led to great progress in understanding the interactions between these two cell types, showing for instance that the axolemma contains a signal that could promote Schwann cell proliferation (Wood and Bunge, 1975; Salzer *et al.*, 1980). This was subsequently identified as neuregulin-1 (Morrissey *et al.*, 1995). The type III neuregulin-1 isoform (TIIINRG1) is expressed on the axolemma of large diameter axons and signals in a juxtacrine manner (via ErbB 2/3 heterodimers expressed by Schwann cells) to promote axon ensheathment and myelination (Garratt *et al.*, 2000; Michailov *et al.*, 2004; Taveggia *et al.*, 2005). Co-cultures recapitulate many of the sequential features of myelination observed *in vivo*, including Schwann cell alignment to axons, ensheathment of the axon by the Schwann cell process and finally membrane wrapping and compaction. The intercellular interactions between axons and Schwann cells during myelination result in the formation of axonal domains with specialised molecular constituents (Rasband and Peles, 2016). For instance, voltage gated sodium channels (VGSCs) are clustered at the node of Ranvier, flanked by the paranodes where tight adherence of the Schwann cell paranodal loops to the axon act as a diffusion barrier, separating VGSCs from potassium channels in the juxtaparanode.

Human induced pluripotent stem cells (iPSCs) are increasingly used in model systems of neurological disorders (Sandoe and Eggan, 2013; Corti *et al.*, 2015). They facilitate the study of human proteins in their normal cellular context, and, through the reprogramming of patient-derived samples, allow the impact of mutations to be investigated within a relevant polygenetic background. They can be used for pharmacological studies, toxicity screening, to investigate immunopathological mechanisms, and to assess the reactivity of cells/antisera towards human antigens. Culture protocols have advanced such that instead of focussing on single cell types it is possible to investigate intercellular interactions and more complex tissues. This approach has been used to investigate central myelin (Czepiel *et al.*, 2015) but has not previously been used to study axoglial signalling and myelination in the peripheral nervous system. We report here that sensory neurons differentiated from human iPSCs can be effectively and reliably myelinated by rat Schwann cells. These myelinating co-cultures display the expected features of axonal and Schwann cell specialization.

In inflammatory neuropathies such as Guillain Barré syndrome (GBS) and chronic inflammatory demyelinating polyneuropathy (CIDP), axoglial proteins and glycolipids are important targets for the underlying autoimmune process (Willison and Yuki, 2002; Devaux *et al.*, 2012). In a subset of patients, neuropathy-associated antibodies specifically target neo-epitopes formed by *cis* and *trans* interactions between different axonal and glial molecules (Querol *et al.*, 2013; Rinaldi *et al.*, 2013). Recreating these interactions in solid phase systems has proved difficult. Problems with existing assays are further evidenced by the inconsistent relationship between antibody binding *in vitro* and pathogenic effects *in vivo* (Greenshields *et al.*, 2009). Anti-disialosyl ganglioside antibodies, which bind to alpha2-8 linked disialic acid residues (as found in GD3, GD1b, GT1b and GQ1b), are associated with predominantly sensory and/or ataxic neuropathies. Disialosyl antibodies of the IgM class are a defining feature of CANOMAD (chronic ataxic neuropathy with ophthalmoplegia M-protein, cold agglutinins and anti-disialosyl antibodies). This neuropathy shows both axonal and demyelinating features on electrophysiology (Willison *et al.*, 2001). Disialosyl antibodies of the IgG class have been found in acute sensory neuropathies, some with unequivocal electrophysiological evidence of demyelination in the form of temporal dispersion (Wicklein *et al.*, 1997; Miyazaki *et al.*, 2001). However, immunization of rabbits with GD1b that raised IgM and IgG antibodies induced a sensory neuropathy characterized pathologically by downregulation of the neurotrophin-3 receptor, trkC, and by the degeneration of sensory axons and DRG neurons (Kusunoki *et al.*, 1996; Hitoshi *et al.*, 1999). In this report, we exploit the myelinating co-cultures to show that both mouse IgG and human, neuropathy-associated IgM type anti-disialosyl antibodies bind to human iPSC-derived sensory axons at the nodal axolemma and, in addition to inducing acute complement mediated axonal degeneration, have deleterious, complement-independent effects on myelination.

## Materials and methods

### Induced pluripotent stem cell generation

NHDF1 (from 44-year-old female) was reprogrammed with Yamanaka retroviruses *SOX2, KLF4*, *OCT3/4*, *c-MYC* and *NANOG* (Takahashi *et al.*, 2006), and has previously been described (Hartfield *et al.*, 2014). AH017-7 (from 67-year-old female) has also previously been described (Handel *et al.*, 2016) and was reprogrammed using the SeVdp(KOSM)302L Sendai virus system, containing genes for *KLF4*, *OCT3/4*, *SOX2* and *c-MYC*, expressed from a single transcript and packaged into a single Sendai virus to ensure consistency of gene dosage ratio (Nishimura *et al.*, 2011, 2013). AD2-1 (from 51-year-old male) was reprogrammed using the CytoTune™-iPS Reprogramming Kit (ThermoFisher) and will be described in an upcoming publication (Buskin *et al.*, in press). The fibroblasts to generate AD2-1 were obtained from a commercial source (Lonza, CC-2511). Further details are supplied in the Supplementary material.

The iPSC line AD2-1 were obtained through the IMI/EU sponsored StemBANCC consortium via the Human Biomaterials Resource Centre, University of Birmingham, UK (http://www.birmingham.ac.uk/facilities/hbrc).

iPSC lines were also subject to strict quality control checks before the initiation of differentiation. Quality control details for AD2-1 are explained in van de Bunt *et al.* (2016). Briefly, this included tests for Sendai virus clearance, FACS for pluripotency markers, genomic integrity checks and embryoid body tri-lineage differentiation experiments. Cells are also confirmed as negative for *Mycoplasma* before cryopreservation.

### Induced pluripotent stem cell maintenance and differentiation

iPSCs were defrosted, plated onto Matrigel^®^ coated plastic and maintained in mTeSR1 medium, which was exchanged daily. iPSCs were passaged when 90% confluent onto Matrigel^®^ coated plasticware using TrypLE express (ThermoFisher). Rho-associated, coiled-coil containing protein kinase (ROCK) inhibitor (ScienCell) (10 μM) was included in the medium for 24 h after each passage. Prior to the start of differentiation, iPSCs were plated onto Matrigel^®^ coated 6-well plates. Twenty-four hours later, the medium was exchanged from mTeSR1 to mouse embryonic fibroblast conditioned medium (ScienCell) supplemented with 10 ng/ml human recombinant FGF2. Cells were allowed to expand in MEF-conditioned medium until ∼50% confluent, at which time differentiation was started according to Chambers *et al.* (2012) (detailed differentiation protocol in Supplementary material). This differentiation resulted in a pure neuronal culture with extensive aborized neurites by 2–3 weeks after the end of the small inhibitor stage.

### Schwann cell harvesting and culture

All work using animals conformed to UK Home Office legislation (Scientific Procedures Act 1986). Schwann cells were harvested from the sciatic nerve and brachial plexus of postnatal Day 3 or 4 rats. Rat Schwann cells are used as well established culture protocols exist and they can be successfully expanded several times *in vitro* in order to create cryopreserved stocks. Nerves were enzymatically digested with a collagenase (Sigma, 3 mg/ml) and dispase II protease (Sigma, 2.5 mg/ml) incubation for 1 h at 37°C. Nerves were gently triturated using a P1000 pipette tip and plated onto PDL/laminin coated plastic in Schwann cell expansion medium [DMEM/F12 (ThermoFisher), 10% foetal bovine serum (ThermoFisher), 200 ng/ml NRG1-β1 EGF domain (R&D Systems), 10 ng/ml NGF (recombinant-murine, Peprotech), 4 µg/ml forskolin (Sigma)]. Cells were serially treated with 5–10 μM araC to eliminate fibroblasts. After approximately four passages, each time doubling the growing area, cells were frozen in a Mr Frosty freezing container (ThermoFisher), and stored in liquid nitrogen.

### Establishing the myelinating co-cultures

To establish the co-cultures, a frozen vial of Schwann cells was thawed in warm water, centrifuged in phosphate-buffered saline (PBS) and resuspended in Schwann cell basal medium [DMEM/F12 (ThermoFisher), 5 µg/ml insulin (Sigma), 100 µg/ml transferrin (Millipore), 25 ng/ml NGF (recombinant-human, Peprotech), 25 ng/ml Selenium (Sigma), 25 ng/ml thyroxine (Sigma), 30 ng/ml progesterone (Sigma), 25 ng/ml triiodothyronine (Sigma), 8 µg/ml putrescine (Sigma)]. The medium on the neuronal cultures was changed from N2 to Schwann cell basal medium. Schwann cells (25 000) in a 25 μl droplet were slowly pipetted onto a single coverslip containing the iPSC-derived neurons, with care taken not to touch the coverslip with the pipette tip. The Schwann cell droplet was evenly distributed over the coverslip. Cultures were carefully put back into the incubator to allow Schwann cell adherence. The culture medium was changed 2 days later. At this stage abundant Schwann cells can be observed adhered to the coverslip. An additional Schwann cell basal medium change was performed 4 days after adding the Schwann cells. During the 1-week period when the human iPSC-derived neurons are cultured in Schwann cell basal medium we observe no detrimental effects to the neurons, such as neurite blebbing. Myelination was induced 1 week after Schwann cell addition, by exposing the cells to myelination medium [N2 medium, 1:300 phenol-free Matrigel^®^ (Scientific Laboratory Supplies), 5% charcoal-stripped FBS (ThermoFisher), 25 ng/ml NGF (recombinant-human, Peprotech), 50 µg/ml ascorbic acid (Sigma)]. Medium changes were performed twice weekly from then on.

When optimizing the culture conditions required to establish myelinating co-cultures, we observed that the Schwann cells proliferate and align to the human axons during the week after seeding them onto the neurons; this is aided by keeping the co-cultures in Schwann cell basal medium. However, we noticed that simply supplementing the Schwann cell basal medium with ascorbic acid (as usually performed when culturing primary DRG neurons and Schwann cells together) resulted in only the occasional formation of myelin internodes. Furthermore, after ∼3–4 weeks these cultures gradually began to show signs of a decline in neuronal health, such as neurite blebbing. We therefore adapted commonly used protocols when generating myelinating co-cultures using primary rodent cells, by using myelination medium that is largely formulated to encourage the growth and wellbeing of the neurons. We found that using the neuronal medium, with the addition of Matrigel^®^and ascorbic acid greatly improved both neuronal health, alignment by Schwann cells and the levels of myelination. This adaptation in medium allowed us to establish healthy and stable myelinating co-cultures between rat Schwann cells and human iPSC-derived neurons that could be kept for many months without signs of a gradual decline in cellular health.

### AAV transduction

For AAV8-Nrg1 type-III production, rat NRG1 type-III was subcloned into an AAV vector. Type-III-β1a constructs were a gift from Dr Douglas L. Falls (Emory University, Atlanta, USA) (Wang *et al.*, 2001). A 2.3kb EcoNI/PsiI insert was cloned from the original PcDNA3.1 vector into the EcoR1 site of a pAAV-CAG vector. The AAV viral packaging, CsCl purification and viral genome titration were performed by Vector Biolabs.

Neuronal cultures were transduced 3 weeks after plating neurons onto coverslips. After infection, neurons were left for 7 days before the addition of Schwann cells. The stock virus was diluted in N2 medium to the correct multiplicity of infection (MOI) based on 25 000 cells per coverslip. N2 medium containing the AAV was then transferred to the neuronal cultures, and left for 3 days. An N2 plus neurotrophins medium change was performed on Days 3 and 5 after infection, and Schwann cells were added on Day 7. Co-cultures were then kept in Schwann cell basal medium for 7 days, before inducing myelination (as described above).

### Immunocytochemistry and confocal imaging

For immunocytochemistry, coverslips were transferred to PBS, and fixed in 1% paraformaldehyde for 20 min. Cells were washed three times in PBS and then permeabilized in ice cold methanol for 20 min. Following three PBS washes, cells were blocked [5% donkey serum, 0.3% milk powder, 1% bovine serum albumin (BSA), 0.1% sodium azide, 1% dimethyl sulphoxide (DMSO)], washed in PBS, and incubated with the primary antibody overnight at 4°C. Cells were then washed with PBS, and incubated with the secondary antibody for 2 h at room temperature. The secondary antibody was washed off with PBS, and the coverslips were mounted onto Superfrost Plus microscope slides (Thermo Scientific) in Vectashield mounting medium (Vector Laboratories). Primary antibodies used and their dilations are indicated in the Supplementary material. Secondary antibodies used were Alexa Fluor 568, 546, 488, 405 or Pacific Blue.

Myelination was quantified via confocal microscopy using systematic random sampling to ensure objective sampling across the coverslip. See Supplementary material for additional detail.

NF200-positive surface area was taken as a measurement of axon area, while myelin basic protein (MBP) positive surface was used as a measurement of myelin area. Since measuring MBP area was a novel approach to quantify myelination, we cross-validated our results by comparing them to the most commonly used method (myelin internode count). We counted the number of internodes and quantified the myelin area in 18 separate myelinated co-culture coverslips within a single neuronal differentiation and found both set of results were highly correlated (Pearson’s R; r = 0.962, *n* = 18, *P* < 0.001; Supplementary Fig. 1). Hence, measurement of MBP surface area was used to quantify myelination, as it provided the more time efficient method. When comparing between conditions, the proportion of the total axonal area covered by myelin (MBP area/NF200 area) was calculated, and expressed as a fold change from control. To acquire cell numbers, DAPI positive nuclei were quantified using ‘Particle Analyser’ provided by ImageJ software.

### Pharmacological blockade of NRG1-ErbB signalling

Pharmacological inhibition of ErbB/NRG1 signalling was performed using the selective and irreversible ErbB inhibitor, PD168393 (7 and 70 nM, reconstituted in DMSO, Calbiochem), and the β-secretase inhibitor IV (50 and 500 nM, reconstituted in DMSO, Calbiochem) that binds to BACE1 active site, blocking its proteolytic activity. Compounds were included in the cell culture myelination medium at all medium changes, at the stated concentrations. Control conditions included the vehicle of the compound (DMSO) at the same concentration. Cells were cultured for 4 weeks with twice weekly medium changes before fixation and image processing.

### Transmission electron microscopy

Cells adhered to coverslips were fixed, dehydrated, infiltrated with epoxy resin and embedded into Agar100 resin. The coverslip was snapped off, leaving the cells embedded as a monolayer on the surface of the block. Ultrathin sections were cut, mounted, post-stained and imaged. Detailed instructions can be found in Supplementary material.

### Anti-disialosyl antibody treatment

The mouse monoclonal IgG antibodies MOG12 and EG4 were produced and purified from hybridomas cloned by limiting dilution after GT1b or GQ1b ganglioside immunization (respectively) of GD3-synthase deficient mice, as previously described (Boffey *et al.*, 2005). The human monoclonal IgM antibody HA1 was cloned after EBV transformation of peripheral blood mononuclear cells taken from a patient with CANOMAD, as previously described (Willison *et al.*, 1996). All of these anti-ganglioside antibodies cross-react with structurally similar gangliosides containing disialosyl groups, including GD1b, GD3, GT1a, GT1b and GQ1b. Herein, they are referred to as ‘anti-disialosyl’ antibodies to reflect this reactivity. Normal mouse IgG was obtained from Abcam. To identify topographical binding patterns, the culture media of mature co-cultures were supplemented with anti-disialosyl antibodies at 100 μg/ml and incubated at 37°C for 16 h. To assess the effects of complement, culture media were subsequently supplemented with 20% normal human serum and re-incubated at 37°C for 2 h. Immunocytochemistry and confocal imaging were then performed, as described above. To assess their effects on myelination, anti-disialosyl antibodies were added to every change of medium for the first 4 weeks after myelination was induced, at both low (10 μg/ml) and high (EG4 40 μg/ml, MOG12 and HA1 50 μg/ml) concentrations, without the addition of normal human serum. Experiments were repeated with three different iPSC differentiations. Controls were treated with myelination medium only, or with identical concentrations of normal mouse IgG. After 4 weeks, cells were fixed, the entire axonal field was imaged, and the proportion of the NF200 positive axonal area covered by MBP positive myelin was calculated as before.

To assess effects on established myelin, co-cultures aged 9 to 12 months from three different iPSC lines were used. At baseline, myelination medium was supplemented with fluoromyelin red (Fisher Scientific) at a 1:300 dilution, and incubated at 37°C for 16 h. The extent of baseline myelination was recorded by imaging live cells over a 2688 µm × 2688 µm centred grid at 10 × objective magnification. Images took less than 3 min to acquire per coverslip. Automated stage movement with defined coordinates were used to allow serial imaging of the same areas. After baseline imaging, myelination medium was replaced. From this point on, the human IgM HA1 was added to each change of medium for the next 4 weeks. Control coverslips were treated with standard myelination medium only. After 4 weeks, cultures were fixed and axon and myelin coverage was assessed by immunofluorescence with NF200 and MBP primary antibodies, as before. To identify areas of demyelination, the fluoromyelin image from baseline was overlain with the MBP channel image at 4 weeks, in which the background had been made transparent. The area of fluoromyelin signal (at baseline) without a corresponding MBP signal (after 4 weeks) was calculated using ImageJ, and expressed as a proportion of the total fluoromyelin area at baseline. Areas of ongoing demyelination, identified by ‘fragmented’ MBP staining replacing linear fluoromyelin staining, were also imaged at higher power (63× objective) to assess the integrity of demyelinated axons.

### Statistics

Statistical analysis was performed using IBM SPSS Statistics for Mac (Version 22). Student’s *t*-test was used for comparison of two groups, one-way ANOVA using Tukey *post hoc* correction was used for more than two groups. Correlation was analysed using Pearson’s R. Results are reported as mean ± SEM. Asterisks indicate the level of significance (^***^P < 0.001, ^**^P < 0.01, *P < 0.05).

## Results

### Alignment and myelination of human induced pluripotent stem cell-derived sensory neurons by rat Schwann cells

**Figure 1:**
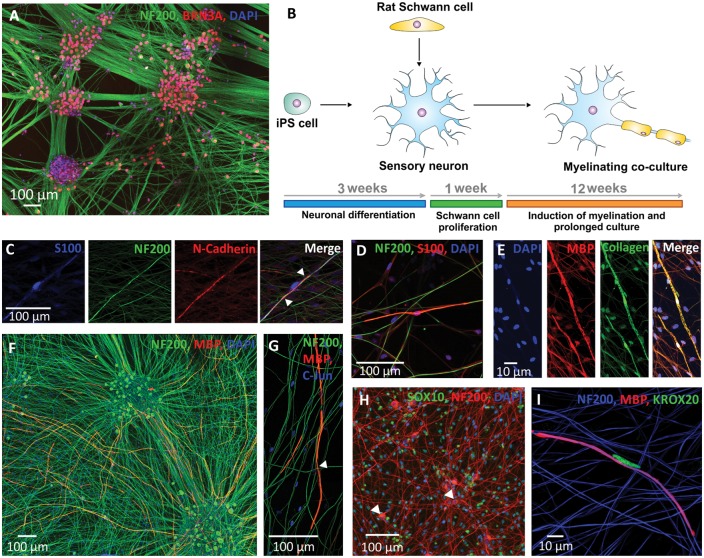
**Rat Schwann cells align and myelinate human iPSC-derived sensory neurons.** (**A**) Sensory neurons can be differentiated from iPSCs, identified through BRN3A positive nuclei, and these grow extensive NF200 immunoreactive neurite processes. (**B**) Schematic showing the establishment of the myelinating co-cultures. (**C**) As rat Schwann cells migrate and come into contact with human axons, N-cadherin expression is upregulated at the interface between the axon and Schwann cell (arrowheads). (**D**) The S100 immunoreactive Schwann cell tightly aligns to the human axon. (**E**) Collagen expression is upregulated as the basal lamina forms around the Schwann cell/axon complex. (**F**) MBP positive internodes are abundant throughout the co-cultures after 6 weeks of myelination. (**G**) Myelinating Schwann cells do not express c-Jun (arrowhead highlights a c-Jun negative nucleus) in contrast to non-myelinating Schwann cells. (**H**) Rat Schwann cells are SOX10 positive, whereas the neurons (NF200 positive, arrowheads) do not express SOX10. (**I**) Myelinating Schwann cells express KROX20.

We found that the key step in promoting myelination in co-cultures of human iPSC-derived sensory neurons and rat Schwann cells was to use a neuronal base medium with Matrigel^®^ in the myelinating medium stage rather than a Schwann cell basal medium. This gave rise to long-lasting stable co-cultures in which neuronal health was maintained and a much greater efficiency of myelination. To investigate how human iPSC-derived sensory neurons interacted with primary rat Schwann cells, co-cultures were investigated for canonical markers of the Schwann cell-axon relationship. Initially, sensory neurons were first differentiated using the previously published protocol (Chambers *et al.*, 2012). These neurons grew extensive arborized neurites, and >90% of cells were positive for the sensory neuron transcription factor BRN3A (Fig. 1A). After allowing the neurons to mature and grow widespread neurite processes for 2 weeks, rat Schwann cells were added in suspension, these adhered to the coverslip and were allowed to migrate and come in contact with axons for 1 week before myelination was induced (schematic and timeline of co-culture shown in Fig. 1B). In that time, alignment and Schwann cell-axon interaction was observed by the expression of N-cadherin at the cell-to-cell interface (Fig. 1C, arrowheads), as has been described in primary co-cultures of DRGs and Schwann cells (Wanner and Wood, 2002). Staining the Schwann cell cytoplasm with an antibody directed against S100 revealed the typical bipolar Schwann cell morphology that was closely aligned to the NF200 positive axon of the sensory neuron (Fig. 1D). As the tightly associated rat Schwann cell begins to form the axon/Schwann cell complex before the onset of myelination, increased levels of extracellular matrix are observed (collagen IV), indicating the beginning of basal lamina formation (Fig. 1E). The first MBP positive internodes were observed after ∼2 weeks of ascorbic acid supplementation, and gradually increased over time in culture (Fig. 3). After 6 weeks of myelination, abundant MBP positive internodes are detected throughout the culture (Fig. 1F). On a transcriptional level, Schwann cells followed a typical transition into a mature myelinating state; basal expression of the transcription factor SOX10 was present in all Schwann cells, but not in neuronal nuclei (arrowheads, Fig. 1H). Upon induction of myelination, rat Schwann cells associated with MBP positive internodes showed strong upregulation of KROX20 (Fig. 1I), while the expression of c-JUN was not detected in these cells (Fig. 1G, arrowhead depicts a Schwann cell nucleus associated with an MBP positive internode that is negative for c-JUN), consistent with the cross antagonistic relationship between these transcription factors (Parkinson *et al.*, 2008). Taken together, these myelinating co-cultures of rat Schwann cell and human iPSC-derived sensory neurons express the canonical markers of an axoglial relationship, and we demonstrate for the first time that there is successful cross-species signalling in order to initiate alignment, basal lamina formation and myelination.

### Node of Ranvier formation and maturation of the myelin sheath

**Figure 2:**
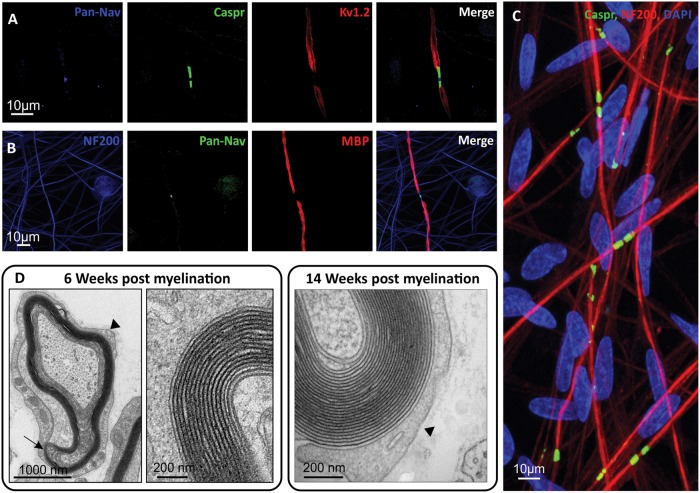
**Nodes of Ranvier and the basal lamina successfully form in co-cultures.** (**A**) Clustering of voltage gated sodium channels at the node, Caspr at the paranode and K_v_1.2 at the juxtaparanode is detected after 4 weeks of myelination. (**B**) Sodium channels cluster between the MBP positive internodes. (**C**) Abundant Caspr positive paranodes are detected after 6 weeks of myelination. (**D**) At the electron microscopy level, the basal lamina can be observed (arrowheads) as well as individual myelin wraps. Not all the myelin wraps are tightly compacted at 6 weeks (arrow), whereas after 14 weeks of myelination, the number of wraps has increased and are tightly compacted.

We detected characteristic nodal structures in our 6-week myelinated co-cultures using antibodies directed against proteins localized to the specialized axonal domains that form at the node of Ranvier, the paranode and the juxtaparanode. Caspr (contactin-associated protein)-positive paranodes encompassed dense clusters of voltage gated sodium channels at the nodes (using an antibody that recognizes a sequence from the intracellular III-IV loop present in all vertebrate Na_v_ channels) (Fig. 2A and B). The shaker type voltage gated potassium channel (K_v_1.2) was localized in adjacent juxtaparanodes (Fig. 2A). Nodes of Ranvier were abundantly detected throughout the culture at this time point, with the occasional heminode also present (Fig. 2C).

Myelin sheath ultrastructure was viewed with electron microscopy at 6 and 14 weeks post-myelination. At this resolution individual myelin wraps and the basal lamina around the Schwann cell/axon complex could be observed (arrowheads, Fig. 2D). At the earlier time point, several axons were observed where the myelin wraps were not compacted (arrow Fig. 2D). Increasing numbers of myelin wraps and a more compacted myelin structure were observed at the later time point indicating the typical maturation of the myelin sheath (Fig. 2D).

### Myelination increases over time

**Figure 3:**
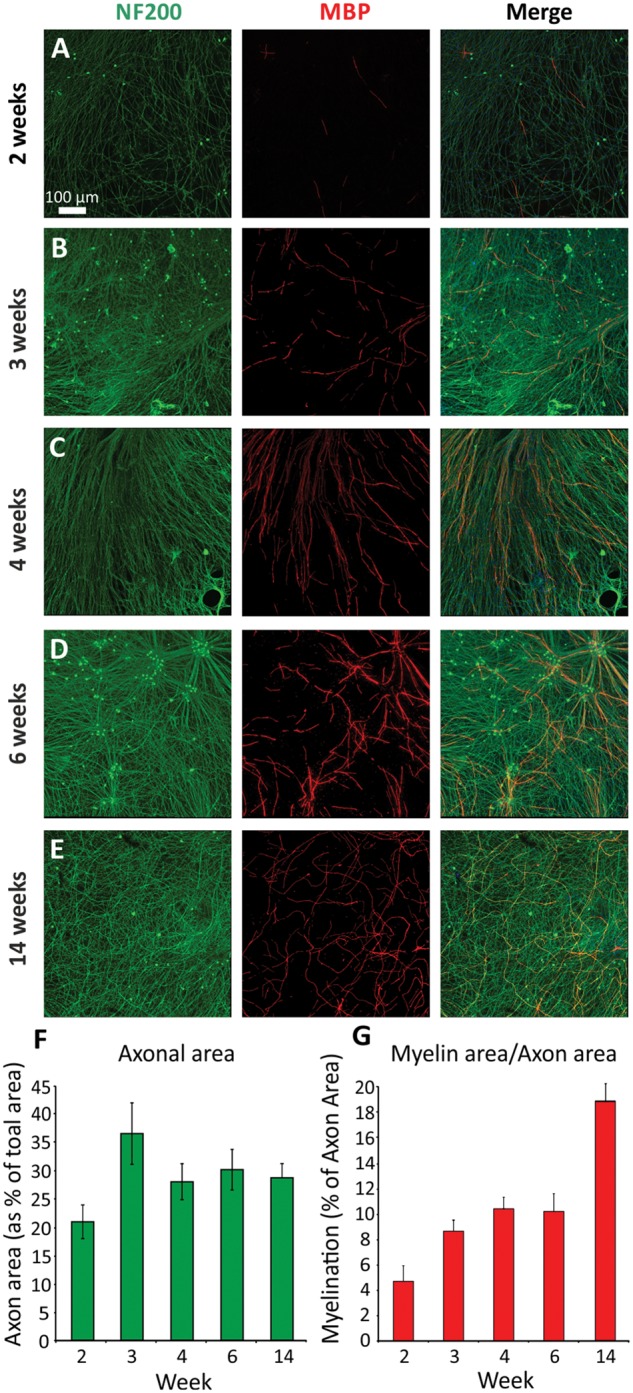
**Myelination increases over time in culture.** Cultures were fixed and immunocytochemistry performed at (**A**) 2, (**B**) 3, (**C**) 4, (**D**) 6 and (**E**) 14 weeks post-induction of myelination. (**A**) The first MBP-positive internodes can be detected at ∼2 weeks. Myelination was quantified (**G**) as a proportion of NF200-positive staining (**F**). Levels of myelination steadily increase over time, whereas axonal area remains unchanged from 4 weeks.

To investigate the time course of myelination in the co-cultures, myelin coverage was quantified at 2, 3, 4, 6 and 14 weeks post-induction. Representative images used for the quantification are illustrated in Fig. 3(A–E). As expected, an overall significant increase in the quantity of myelin was observed over time [Fig. 3G, ANOVA, *F*(4,17) = 9.616, *P* < 0.001], however the axonal coverage did not significantly change over time [Fig. 3F, ANOVA, *F*(4,17) = 2.735, *P* > 0.05], indicating that the increase in myelin is not simply due to greater axonal outgrowth. Furthermore, these cultures are extremely stable, and have been maintained for over 9 months with no loss of myelin or neuron integrity (Supplementary Fig. 2).

To assess the levels of myelination across different iPSC lines, we differentiated three separate control lines to sensory neurons and co-cultured them with rat Schwann cells as described in the methods. All three iPSC-derived neuronal cultures were successfully myelinated and the levels of myelination were very similar between cell lines (Supplementary Fig. 3) demonstrating the reproducibility of this co-culture system.

### Overexpression of NRG1 type III influences levels of myelination

**Figure 4:**
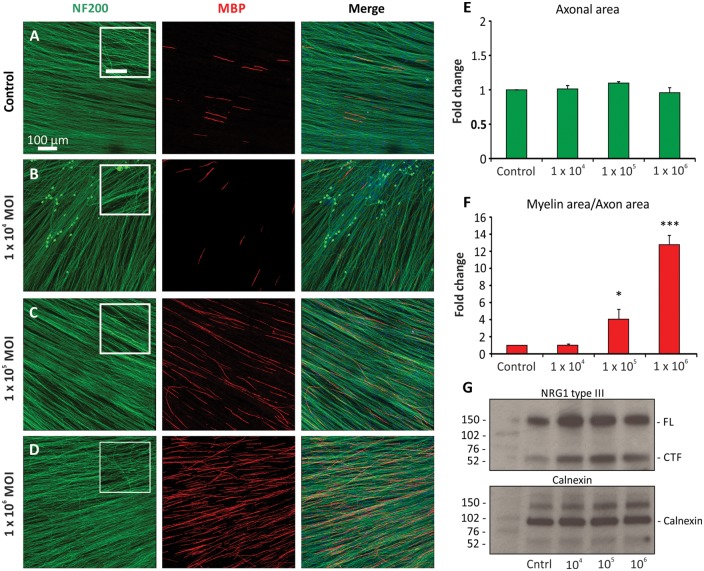
**Overexpression of TIIINRG1 significantly increases myelination in the co-cultures.** (**A–D**) Human iPSC-derived neurons were transduced with an AAV containing a TIIINRG1 construct at a range of MOI’s. Cultures were fixed for immunocytochemistry or lysed for protein extraction at 4 weeks post-induction of myelination. (**E**) Viral transduction had no effect on axonal outgrowth or morphology, with no visible signs of neurite blebbing (insets in **A–D**). Scale bar = 50 μm. (**F**) Myelination was similar in (**A**) untransduced and (**B**) 1 × 10^4^ MOI conditions, whereas a significant and dose dependent increase in myelination was observed in (**C**) 1 × 10^5^ (*P < 0.05) and (**D**) 1 × 10^6^ (^***^P < 0.001) conditions. (**G**) In the transduced neurons, western blot shows the increase in both the full-length (FL) (135 kDa) and cleaved-terminal fragment (CTF) (60 kDa) of TIIINRG1 compared to untransduced control. The calnexin control demonstrates an even protein loading across conditions.

TIIINRG1 expression at the axonal surface has been established as one of the key regulators of myelination. To investigate the role of TIIINRG1 in the present co-culture system, human iPSC-derived neurons were transduced with an AAV containing a TIIINRG1 construct at a range of MOIs (1 × 10^4^ to 1 × 10^6^, Fig. 4B–D). Viral transduction was well tolerated by iPSC-derived neurons, as no difference in axon area (Fig. 4E) [ANOVA, *F*(3,17) = 1.103, *P* > 0.05] or visible signs of cellular stress were observed across different conditions (Fig. 4A–D). Neuronal overexpression of TIIINRG1 type caused a significant increase in the formation of myelin at 4 weeks (Fig. 4F) [ANOVA, *F*(3,17) = 47.87, *P* < 0.001], with 4.8-fold and 13-fold increase with 1 × 10^5^ and 1 × 10^6^ MOI, respectively. Full-length (FL) and the cleaved-terminal fragment (CTF, formed following proteolytic processing) of TIIINRG1 protein expression was increased in the 1 × 10^5^ and 1 × 10^6^ MOI condition compared to untransduced basal expression (Fig. 4G), which significantly increased the levels of myelination in both conditions (Fig. 4F). Interestingly, viral transduction with 1 × 10^4^ MOI concentration led to some increase in the protein levels of both the full-length and cleaved-terminal fragment (Fig. 4G), without influencing the levels of myelination (Fig. 4F) suggesting a possible threshold effect, furthermore assessing total NRG1 levels is only a partial reflection of TIIINRG1 protein at the axoglial interface.

### Pharmacological blockade of NRG1-ErbB signalling leads to impaired myelination

**Figure 5:**
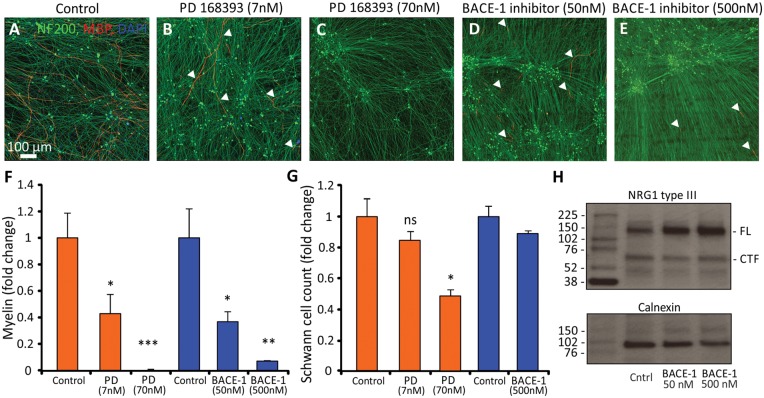
**Pharmacological blockade of NRG1/ErbB signalling impairs myelination in the human/rat co-cultures.** Irreversible blockade of ErbB receptors with PD168393 at (**B**) 7 nM and (**C**) 70 nM dose-dependently reduces the formation of MBP positive internodes compared to (**A**) control cultures. Inhibition of the β-secretase protease (BACE1) with (**D**) 50 nM and (**E**) 500 nM also results in a dose-dependent reduction in the formation of myelin. The few remaining myelin internodes are indicated in **B**, **D** and **E** with arrowheads. (**F**) Quantification of the myelin levels relative to an internal control. (**G**) Schwann cell numbers are reduced only with the highest concentration of PD168393. (**H**) Treatment of iPSC-derived sensory neurons with BACE1 results in an accumulation in full-length (FL) TIIINRG1, and a reduction in the cleaved-terminal fragment (CTF).

Neuregulins belong to the family of EGF domain-containing proteins and interact with and activate receptor tyrosine kinases of the ErbB family expressed by Schwann cells (ErbB2 and ErbB3). The selective and irreversible ErbB inhibitor PD168393 was used to block ErbB receptors and prevent TIIINRG1 signal transduction*.* As expected, a dose-dependent reduction in myelination was detected in the presence of PD168393 inhibitor (Fig. 5B, C and F) [ANOVA, *F*(2,14) = 14.87, *P* < 0.001]. TIIINRG1 requires proteolytic cleavage by proteases to expose its active EGF-domain (Hu *et al.*, 2006; Willem *et al.*, 2006; Fleck *et al.*, 2016). The cell-permeable isophthalamide that binds to BACE-1 active site was used to block its proteolytic activity. The presence of the BACE1 inhibitor significantly reduced myelination when compared to the control conditions (Fig. 5D–F) [ANOVA, *F*(2,13) = 7.421, *P* < 0.01]. These results indicated that interference with NRG1-ErbB signalling reduced myelination in our co-culture system. Neither the ErbB inhibitor [ANOVA, *F*(2,14) = 2.311, *P* > 0.05] nor the BACE1 inhibitor [ANOVA, *F*(2,13) = 1.452, *P* > 0.05] had a measurable effect on axonal density (Fig. 5B–E). To assess whether the pharmacological blockade of NRG/ErbB signalling led to a reduction in Schwann cell proliferation, we quantified the number of DAPI positive Schwann cell nuclei in our cultures. Only the highest concentration of PD168393 resulted in a reduction in Schwann cell numbers (Fig. 5G), consistent with the fact that NRG1 promotes stem cell proliferation. When we assessed the protein levels of the full-length and cleaved-terminal fragment of TIIINRG1 after neurons have been treated with the BACE1 inhibitor, we found that there was a reduction in the amount of cleaved-terminal fragment, and an increase in the full-length levels with both concentrations of BACE1 inhibitor. This corresponds to the inhibition of the proteolytic cleavage of full-length TIIINRG1, resulting in an accumulation of the full-length and a reduction in the cleaved-terminal fragment. Similar results were observed when assessing full-length and cleaved fragment levels in the *Bace1*^-/-^ mouse (Hu *et al.*, 2006; Willem *et al.*, 2006).

### Anti-disialosyl antibodies impair myelination

**Figure 6:**
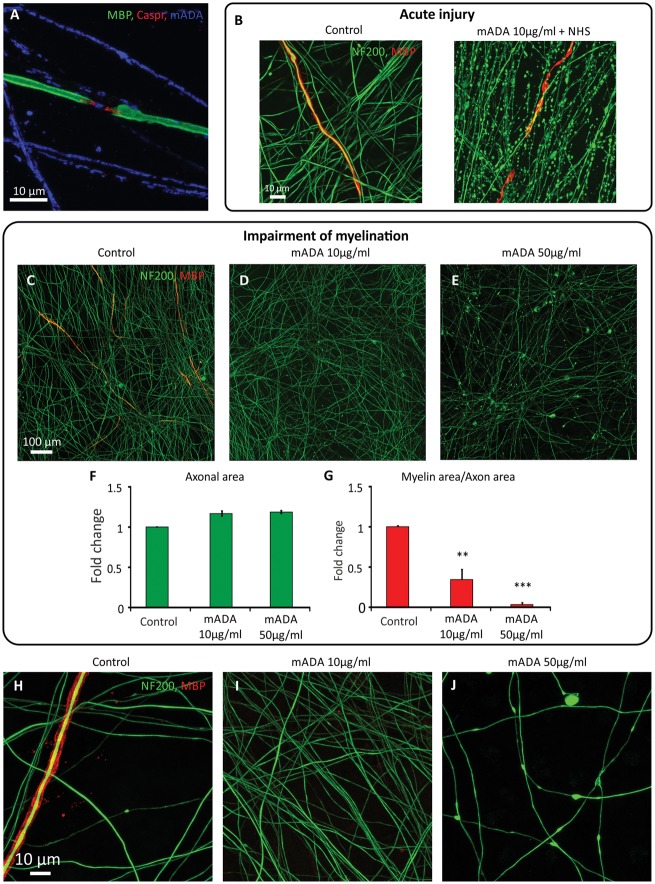
**Mouse IgG anti-disialosyl antibodies cause acute, complement dependent axonal degeneration while prolonged application without a source of complement impairs myelination.** (**A**) Both mouse IgG3 antibodies (mADAs, example shown is EG4, blue) bind to unmyelinated axons and the nodal axolemma, but do not cross the paranodal junction (marked by Caspr, red). (**B**) With the additional provision of normal human serum (NHS) as a source of complement, axonal degeneration can be observed after 2 h. (**C–E** and **G**) Prolonged culture with mouse anti-disialosyl antibodies without a source of complement significantly reduces the extent of myelination at 4 weeks at both low (10 μg/ml, ^**^P < 0.01) and high (40 μg/ml EG4, 50 μg/ml MOG12, ^***^P < 0.001) antibody concentrations. (**F**) Axonal area in antibody-treated cultures is not reduced over this time period. Graphs show pooled data from three separate experiments using both anti-disialosyl antibodies (EG4 and MOG12). Example images were treated with MOG12 (‘mADA’) or myelination medium only (‘Control’). (**H–I**) Axonal morphology is no different from controls after 4 weeks of continuous incubation with low dose (10 μg/ml) mouse anti-disialosyl antibodies without NHS, but (**J**) irregular axonal staining and blebbing is seen at 50 μg/ml.

**Figure 7:**
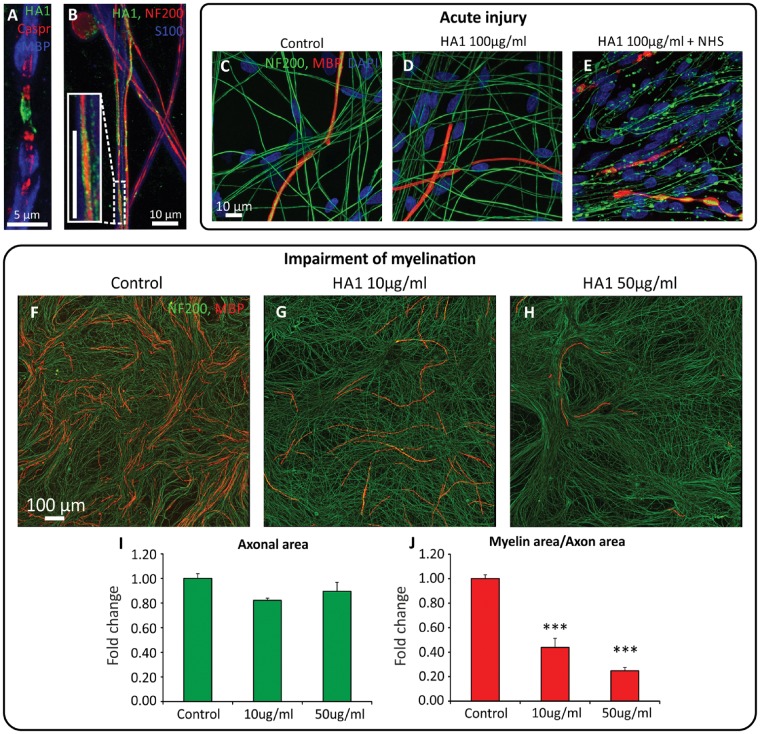
**A CANOMAD associated human IgM anti-disialosyl antibody (HA1) also causes acute, complement dependent axonal degeneration while prolonged application without a source of complement impairs myelination.** (**A**) HA1 (green) binds to nodal axolemma (flanked by Caspr, red) and to (**B**) unmyelinated axons (NF200, red), without any reactivity to Schwann cells (S100, blue). (**C–E**) Short-term incubation with HA1 followed by normal human serum as a source of complement leads to (**E**) acute axonal degeneration. (**F–H**) Prolonged incubation with HA1, without provision of complement, leads to a dose-dependent reduction in myelination over 4 weeks, quantified in (**J**). No significant alteration to (**F–H**) axonal morphology or (**I**) axonal coverage was observed (^***^P < 0.001 versus control).

Gangliosides containing disialosyl moieties are known to be expressed by sensory neurons (Svennerholm *et al.*, 1994). Auto-antibodies directed against these gangliosides are associated with acute (IgG) and chronic sensory/ataxic neuropathies (IgM) with electrophysiological evidence of both axonal degeneration and demyelination (Wicklein *et al.*, 1997; Miyazaki *et al.*, 2001; Willison *et al.*, 2001). We therefore investigated the topographical targets and pathological effects of anti-disialosyl antibodies using two mouse IgG3 monoclonal antibodies (MOG12 and EG4) and the human IgM monoclonal, HA1. After overnight incubation, all antibodies had bound to exposed axolemma (at both the nodes of Ranvier) (Figs 6A and 7A) and unmyelinated axons (Figs 6A and 7B) within the mature co-cultures. Nodal reactivity was sharply delimited by the paranodal junction protein Caspr indicating that tight junctions at the paranode are likely excluding access of these antibodies to the internodal regions (Figs 6A and 7A). We did not observe binding to Schwann cells. After overnight incubation with either mouse or human ADAs at 10 or 100 μg/ml, the addition of normal human serum as a source of complement produced axonal deterioration and myelin blebbing within 2 h (Figs 6B and 7E). These changes were not seen with antibody or normal human serum alone (Figs 6B, and 7C and D). The prolonged addition of the low concentrations of mouse IgG anti-disialosyl antibodies (10 μg/ml), without normal human serum, started at the onset of myelination, significantly reduced the proportion of myelination at 4 weeks (Fig. 6D and G) [*n* = 3, mean (95% confidence interval, CI) reduction versus control = 66% (35% to 96%), *P* < 0.01, one-way ANOVA with Tukey correction], without reducing the total axonal area itself (Fig. 6D and F). Unlike the changes observed following the addition of normal human serum, this condition did not result in axonal degeneration. Likewise, there were no morphological changes in the axons after 4 weeks, when compared to control conditions without antibody (Fig. 6H and I). Prolonged incubation with higher concentrations of mouse IgG ADAs (50 μg/ml) almost entirely prevented myelination [Fig. 6E and G, *n* = 3, mean (95% CI) reduction versus control = 96% (64% to 127%), *P* < 0.001, one-way ANOVA with Tukey correction], but also produced widespread irregularities in the axonal neurofilament-heavy staining (Fig. 6E and J). There was no significant difference between the two different mouse IgG anti-disialosyl antibodies in terms of their observed effects on myelination at either the high or low concentration (both *P* > 0.05, unpaired *t*-test), and these results were pooled for the final analysis. No significant effect on myelination was seen with control antibodies (normal mouse IgG, Abcam) at either high (50 μg/ml) or low (10 μg/ml) concentration (*P* > 0.05, one-way ANOVA with Dunnett correction) (Supplementary Fig. 4).

The prolonged addition of the human IgM monoclonal, HA1, started at the onset of myelination, likewise resulted in a dose-dependent reduction in axonal coverage by myelin at 4 weeks [*n* = 3, mean (95% CI) reduction at 10 μg/ml versus control = 56% (35% to 77%), *P* < 0.001, mean (95% CI) reduction at 50 μg/ml versus control = 75% (54% to 96%), *P* < 0.0001, one-way ANOVA with Tukey correction] (Fig. 7F–J). Neither antibody concentration caused visible changes to axonal morphology (Fig. 7F–H) or a significant change in axonal area [one-way ANOVA with Tukey correction, *F*(2,6) = 0.9833, *P* > 0.05] (Fig. 7I).

### A human anti-disialosyl IgM antibody induces demyelination

**Figure 8:**
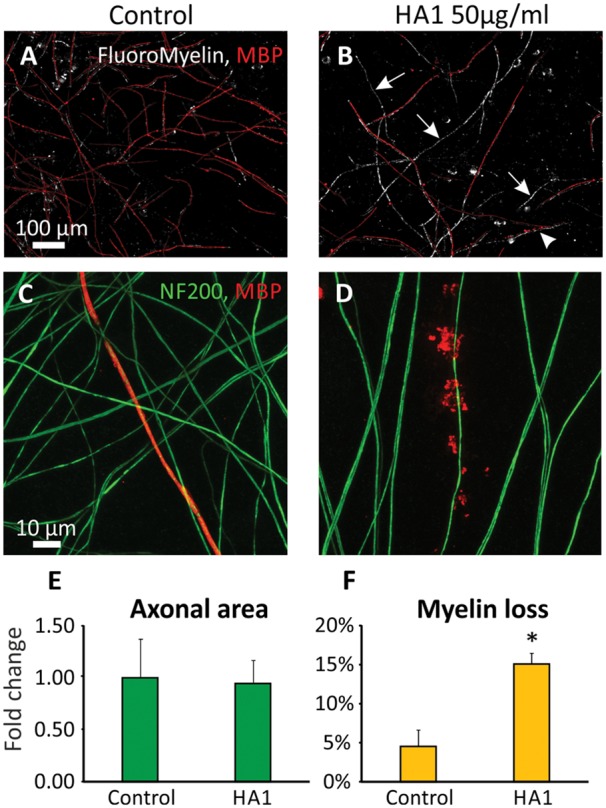
**A CANOMAD associated human IgM anti-disialosyl antibody induces complement independent demyelination without evidence of axonal injury.** (**A** and **B**) Superimposed images of the same field of view of an established myelinating co-culture 4 weeks apart. (**A**) Under control conditions the pattern and extent of myelination at baseline (assessed by FluoroMyelin™ staining, white) was largely maintained after 4 weeks further culture (assessed by MBP immunofluorescence, red). (**C**) Myelin and axons were morphologically normal in these aged co-cultures. (**B**) With prolonged exposure to HA1, numerous internodes present at baseline were no longer apparent after 4 weeks (arrows). In some areas (arrowhead) fragmentation of myelin could be seen. (**D**) At higher power in fixed tissue, myelin degeneration in such areas could be observed without loss of the underlying axon. (**E**) When quantified, there was no significant difference in axon area between HA1 treated and control cultures, but (**F**) the proportion of myelin loss was significantly greater with HA1 (*P < 0.05).

To establish whether ADAs could induce demyelination, in addition to preventing myelination, established co-cultures aged 9–12 months were used. With prolonged exposure to HA1 at 50 μg/ml, numerous myelin internodes present at baseline (revealed by live cell FluoroMyelin™ staining) were absent on repeat images taken after 4 weeks (Fig. 8B). In comparison, under control conditions, the majority of internodes remained stable over the same time period (Fig 8A). Higher power images revealed no differences in axonal morphology between the groups (Fig. 8C and D). Furthermore, in HA1 treated cultures, presumed ongoing demyelination could occasionally be observed, with clumps of disintegrating myelin surrounding intact axons (Fig. 8D). When quantified at 4 weeks, there was no significant difference in axonal area between HA1 treated and control cultures [mean (95% CI) coverage difference for HA1 versus control = 0.05 (−1.24 to 1.13), *P* = 0.9039, unpaired *t*-test] (Fig. 8E). However, the extent of myelin loss (demyelination) was significantly higher in the HA1 group [mean (95% CI) increased myelin loss with HA1 versus control = 10.54% (3.92 to 17.17), *P* = 0.0115, unpaired *t*-test] (Fig. 8F).

## Discussion

Our aim was to establish myelinating co-cultures from iPSC-derived sensory neurons and use them to investigate the physiology and pathology of axoglial interactions within the peripheral nervous system. We found that, surprisingly given the species difference, human iPSC-derived sensory neurons can be reliably and efficiently myelinated by rat Schwann cells. These co-cultures feature the expected markers of a specialized axoglial relationship, including the widespread formation of nodes of Ranvier. The cultures are also extremely stable over time, which is a significant advantage compared to primary myelinating co-cultures, and will enable axoglial physiology and disease pathogenesis to be investigated over long time periods.

iPSCs have become a fundamental tool for disease modelling, drug and toxicology screening and regenerative medicine. With the intrinsic capability of almost limitless self-renewal, the theoretical capacity to differentiate into essentially every cell type, and the potential to model a pathogenic genotype by reprogramming cells from an affected individual, iPSCs have attracted considerable attention (Soldner *et al.*, 2012; Sterneckert *et al.*, 2014; Hockemeyer and Jaenisch, 2016). This led to rapid advances in the field, both in terms of reprogramming methods and in the development of novel and improved differentiation protocols for a large variety of cell types (Mertens *et al.*, 2016). The differentiation protocol for sensory neurons used in the current study is exemplary (Chambers *et al.*, 2012). Using a combination of five small molecular pathway inhibitors, this method produces a sensory neuronal population within 2 weeks at an efficiency of >90%. Further characterization defined these neurons as molecularly and functionally comparable to human sensory neurons derived from mature dorsal root ganglia. The protocol was originally developed with the aim of generating nociceptors, most (but not all) of which have small diameter, unmyelinated axons. However, the neurons generated are heterogenous (Young *et al.*, 2014). We noted approximately a third of neurons generated using this protocol had action potential characteristics of non-nociceptive afferents (unpublished observation) and all axons were immunoreactive for neurofilament heavy chain, a characteristic of large diameter axons. We therefore investigated whether these sensory axons could be myelinated. We observed that iPSC-derived sensory neurons were indeed able to engage in the highly complex cell-to-cell interactions with Schwann cells, and successfully undergo myelination. Direct comparisons of myelination with primary co-cultures of rat DRG neurons with rat Schwann cells have not been conducted however, qualitatively the onset and nature of myelination is very similar. The longevity of our co-cultures is substantially greater than primary rat/rat co-cultures, so whilst similar levels of myelination may be initially observed after several weeks, we observe a progressive increase in myelination over time. Co-cultures could be maintained for >9 months without loss of myelin or axonal integrity (Supplementary Fig. 2), qualifying them for studies that require extremely long timeframes.

The activation of the myelination process (Fig. 1) and the formation of nodal structures (Fig. 2), require sequential and highly complex reciprocal cellular interaction (Salzer, 2015). While many factors play a role in myelination, including cAMP, brain-derived neurotrophic factor (BDNF), Notch 1, cell adhesion molecules, and G-protein-coupled receptors, NRG1-ErbB signalling has been established as a key regulator of this process (Taveggia, 2016). All NRG1 isoforms contain an epidermal growth factor (EGF)-like signalling domain that can act on heterodimeric ErbB2-ErbB3 receptors expressed at the Schwann cell membrane; the membrane-bound type III isoform is thought to be the most important signalling molecule for the regulation of myelination in the PNS (Michailov *et al.*, 2004; Taveggia *et al.*, 2005; Birchmeier and Bennett, 2016). Using an AAV-mediated overexpression system or pharmacological inhibition, we find that TIIINRG1 expressed on human axons could strongly promote myelination. Levels of NRG1 in the peripheral nervous system are regulated by proteolytic cleavage by a range of secretase enzymes, including BACE1 (Hu *et al.*, 2006; Willem *et al.*, 2006; Fleck *et al.*, 2016). The role of NRG1-ErbB signalling in myelination of the rodent peripheral nervous system is well established, and we have now shown that modulating TIIINRG1 expression on human sensory axons also has a key role in regulating myelination. Co-cultures also have potential for drug and toxicology screening (Fig. 5). In the last decade BACE1 has emerged as an important target for experimental therapies in Alzheimer’s disease. In addition to the proteolytic processing of NRG1, BACE1 is one of the β-secretase enzymes required for the endoproteolysis of the amyloid precursor protein (APP), and hence the production of the 42-amino acid long β-amyloid peptide (amyloid-β_42_), widely considered to be neurotoxic and crucial during the early onset of Alzheimer’s disease (Vassar, 2004). As a result, BACE1 inhibitors are currently in human clinical trials (Vassar *et al.*, 2014). Given the profound effect on the myelination of human axons, the potential for BACE1 inhibition to produce deleterious effects on peripheral nerve function is highlighted by this study. This may be a particular problem for patients with pre-existing neuropathies (up to 8% of the elderly population) (Martyn and Hughes, 1997), in which ongoing repair may be critically important. NRG1 is now also being considered as a target for clinical intervention. Human based myelinating co-cultures should prove a useful tool for screening the effects of drugs targeting this signalling pathway.

iPSC-based assays also offer advantages in the investigation of autoimmune diseases. Indeed, non-myelinating cultures of iPSC-derived motor neurons have recently been used to study the immunopathology of multifocal motor neuropathy associated anti-GM1 antibodies (Harschnitz *et al.*, 2016). Dysimmune processes underlying other inflammatory neuropathies are known to target distinct topographical regions of the peripheral nerve, including the myelin sheath and specialized axonal domains formed at the node of Ranvier and flanking paranodal and juxtaparanodal domains (Devaux *et al.*, 2012; Querol *et al.*, 2013). These structures are not formed in pure neuronal cultures, further highlighting the benefits of myelinating co-cultures. Currently, the specific antigen target in many inflammatory neuropathies is elusive and the exact nature of their pathology is uncertain. This is likely to be because solid phase serological assays fail to recapitulate the complex antigen environment created by *cis* interactions within neural membranes, and the *trans* interactions, which characterize axoglial connections and signalling. Such assays provide little to no insight into pathological mechanisms. The co-culture system thus has great promise for strengthening clinical-serological correlations, identifying new antigen targets, establishing pathological mechanisms, and testing therapeutic strategies. A further advantage of this culture system is its stability over time, facilitating the more realistic study of longer term pathological processes. Ganglioside antibodies have previously been shown to cause acute axonal deterioration in the presence of complement (O’Hanlon *et al.*, 2001; McGonigal *et al.*, 2010), which is again confirmed by this study. We have now shown that both mouse- and patient-derived anti-disialosyl ganglioside antibodies disrupt myelination independently of complement. The finding that these antibodies bind to the axolemma and not directly to Schwann cells emphasizes the importance of axon-derived signals in the formation and maintenance of healthy myelin. These observations correlate with the demyelinating electrophysiological pattern seen in some acute sensory neuropathies associated with anti-GD1b antibodies of the IgG class (Wicklein *et al.*, 1997), and with CANOMAD (associated with IgM anti-disialosyl antibodies), which shows both axonal and demyelinating features (Willison *et al.*, 2001). These antibodies not only induce demyelination, but also block *de novo* myelination in this experimental system. Anti-ganglioside antibodies have previously been shown to reduce axonal regeneration in culture (Rozés Salvador *et al.*, 2016), and their persistence in patients with GBS is associated with delayed recovery (Bech *et al.*, 1997). Our observations also suggest that they may impair repair associated remyelination, with potential implications for the optimal timing and duration of immunomodulatory therapy in patients. The anti-disialosyl antibodies in this study also react with GT1b, a known ganglioside ligand for myelin associated glycoprotein (MAG) providing one potential mechanism for altered axoglial interactions (Yang *et al.*, 1996). MAG is involved in the maintenance of myelin, whereas evidence of its role in the initiation of peripheral nervous system myelination is conflicting (Owens *et al.*, 1990; Owens and Bunge, 1991; Montag *et al.*, 1994). Mice with disrupted ganglioside synthesis do however display increased axonal degeneration and demyelination within their peripheral nerves, possibly because the ganglioside-MAG interaction is absent (Sheikh *et al.*, 1999). Whether anti-disialosyl antibodies bound to axonal gangliosides block this specific signal, affect another unidentified pathway, or simply act as a non-specific physical barrier to axoglial interaction, remains to be determined.

In summary, we have developed a myelinating co-culture system using human iPSC-derived sensory neurons and rodent Schwann cells, which faithfully recapitulates the axoglial signalling required for myelination. This co-culture system can be used to screen drugs that may promote or impede myelination and to investigate the immunopathology of the inflammatory neuropathies.

## Supplementary Material

Supplementary Data

## Abbreviations

iPSC: induced pluripotent stem cell
MOI: multiplicity of infection
TIIINRG1: type III neuregulin-1
