# Supplementary material for: Co-cultures with stem cell-derived human sensory neurons reveal regulators of peripheral myelination
Source: Brain. 2017 Feb 15;140(4):898–913. doi: 10.1093/brain/awx012 (PMC5637940; doi:10.1093/brain/awx012)
Supplement: Supplementary Data [file awx012_supp.zip › brain-2016-01328-File017.pdf]

Supplementary figure 1

The number of MBP positive internodes and MBP area (as a proportion of NF200 immunoreactive axons) is highly correlated (Pearson's R; $r = 0.962$, $n = 18$, $P < 0.001$).

Supplementary figure 2

Myelinating co-cultures can be maintained in culture for many months. This image illustrates a 9 month old myelinating coculture. The neurons show no signs of cellular stress such as neurite blebbing and there are abundant MBP positive internodes throughout the culture.

Supplementary figure 3

The levels of myelination are similar across three different iPSC lines. When three different iPSC lines were separately differentiated to neurons and myelinated with rat Schwann cells, the levels of myelination were very similar when quantified at 4 weeks.

Supplementary figure 4

Prolonged (4 week) incubation with control antibodies (normal mouse IgG, nIgG) does not significantly alter either (A) axonal or (B) myelin coverage.
