# Supplementary material for: Co-cultures with stem cell-derived human sensory neurons reveal regulators of peripheral myelination
Source: Brain. 2017 Feb 15;140(4):898–913. doi: 10.1093/brain/awx012 (PMC5637940; doi:10.1093/brain/awx012)
Supplement: Supplementary Data [file awx012_supp.zip › brain-2016-01328-File014.pdf]

Supplementary methods

Ethics statement

Human iPSC lines used in this study were derived from human skin biopsy fibroblasts, following signed informed consent. Three control cells lines were used in this study – AD2-1 (iPSC line 1), AH017-7 (iPSC line 2) and NHDF1 (iPSC line 3). AH017-7 and NHDF1 were reprogrammed with approval from research ethics committee: National Health Service, Health Research Authority, NRES Committee South Central, Berkshire, UK (REC 10/H0505/71).

iPSC reprogramming

CytoTune-iPS Reprogramming Kit (ThermoFisher) was used to generate AD2-1, Reprogramming was performed as directed by the manufacturer's instructions. The CytoTune reprogramming kit contains four Sendai virus-based reprogramming vectors each capable of expressing one of the four Yamanaka factors (*KLF4*, *OCT3/4*, *SOX2* and *c-MYC*). Briefly, after fibroblast transduction with the four Sendai virus-based reprogramming vectors, cells are cultured for 5-6 days, with medium changes every other day (DMEM, high glucose (Sigma), 10% FBS (ThermoFisher), 1% Pen/Strep (100x, ThermoFisher), 200mM L-glutamine (Sigma), 1% non-essential amino acids (ThermoFisher)). The transduced fibroblasts are then passaged using 0.05% Trpsin-EDTA onto pre-prepared feeder layer plates containing mitotically inactivated mouse embryonic fibroblasts (MEF). 3-4 weeks after transduction, colonies should have grown to an appropriate size to allow for manual picking. Using an inverted microscope, a single colony displaying iPSC morphology is cut into 5-6 pieces using a 25 gauge needle, transferred into iPS media (KO-DMEM (ThermoFisher), 25% Knock Serum Replacement (ThermoFisher), 1% nonessential amino acids (100x, ThermoFisher), 200mM L- glutamine (Sigma), 1% Pen/Strep (100x, ThermoFisher), 8 ng/ml human FGF2 (Miltenyi Biotec)) and plated onto pre-prepared MEF plates. Colonies are allowed to attach for 48 hours, and thereafter medium changes are performed daily. iPSCs were adapted to feeder-free conditions onto Matrigel (Scientific Laboratory Supplies)-coated plates in mTeSR1 medium (ScienCell). Bulk passaging was by 0.5 mM ethylenediaminetetraacetic acid (EDTA) to make large-scale, quality-controlled stocks that were cryopreserved in liquid nitrogen. The number of feeder-free passages was kept to a minimum. When selecting iPSCs from frozen stocks for differentiation, vials with the same

passage number were selected for each cell line throughout all experiments performed in this study.

Neuronal differentiation

Briefly, the medium was exchanged to knockout serum replacement (KSR) medium (Knockout-DMEM (ThermoFisher), 15% knockout-serum replacement (ThermoFisher), 1% Glutamax (100x, ThermoFisher), 1% nonessential amino acids (100x, ThermoFisher), 100 μM β-mercaptoethanol (ThermoFisher), 1% antibiotic/antimycotic (100x, ThermoFisher)) containing the SMAD inhibitors –SB431542 (Sigma, 10 μM) and LDN-193189 (Stratech, 100 nM). The medium was gradually transitioned from KSR medium to N2 medium (Neurobasal medium (ThermoFisher), 2% B27 supplement (ThermoFisher), 1% N2 supplement (ThermoFisher), 1% Glutamax (ThermoFisher), 1% antibiotic/antimycotic (ThermoFisher)) over an 11 day period. On day 2, the small molecules - CHIR99021 (Apollo Scientific, 3 μM), SU5402 (R&D Systems, 10 μM) and DAPT (Sigma, 10 μM), were included together with the dual SMAD inhibitors in the medium. Whilst not in the original description by Chambers et al., 2012, we found it optimal to passage the cells on day 2 or 3 of the differentiation, which allowed more growing area for the neurons to develop into. On day 6, the SMAD inhibitors were removed, leaving only the 3 small inhibitors in the medium. On day 11, the now immature neurons were replated using TrypLE (Gibco) onto Matrigel coated coverslips (25,000 per 13 mm coverslip) in 100% N2 medium containing human recombinant NGF, GDNF, BDNF and NT3 (all at 25 ng/ml, Peprotech). CHIR99021 (3 μM) was included in the medium until day 14, and laminin (1 μg/ml, ThermoFisher) was supplemented into the medium from day 20 onwards. Medium changes were performed twice weekly after replating onto coverslips. If required, Cytosine β-D-arabinofuranoside (araC, 2 μM, Sigma) was included in the medium soon after replating to kill the few non-neuronal dividing cells remaining in the culture. AraC was withdrawn from the medium once a pure neuronal culture was obtained, as judged by the absence of morphologically non-neuronal cells on phase-contrast light microscopy. This state was typically achieved 2-3 weeks after replating.

Confocal imaging and quantification of myelination

Zen Black Software (Zeiss, Germany) was used to create a digital diamond shape of 13 points (2 mm horizontal/vertical distance between each point) that was centred on each coverslip. A tile scan (each 1792 µm x 1792 µm in size, using either a 10x or 20x objective) was taken at

each point, resulting in 13 non-overlapping images that equally sampled all quadrants of the coverslip (31.4% of the total area). Images were processed and analysed using ImageJ software (Version 1.48J). A condition-blinded investigator then created binary images for each channel (MBP (Myelin Basic Protein), NF200 (Neurofilament Heavy Chain) & DAPI) by adjusting the threshold to acquire the maximum signal-to-noise ratio, and area measurements for each channel were recorded. The proportion of axonal area covered by myelin was then calculated.

Western blot

Cells were washed with PBS, and then lysed in NP40 buffer. Mechanical dissociation through a 25 gauge needle was used to facilitate breakdown of the cellular material. The lysates were spun at 13,000 rpm for 15 min and the protein concentration of the supernatant was determined using a BCA Protein Assay kit (ThermoFisher). Protein homogenate (30 µg) was loaded on precast 10-14% SDS-polyacrylamide gels (Biorad), and transferred to PVDF membranes (ThermoFisher), blocked in 3% BSA for 1 hour and immunoblotted with antibodies against NRG1 (Neuregulin-1α/β1/2 (C-20) SantaCruz SC-348; 1:500) and Calnexin (Endo Life ADI-SPA-860; 1:10,00). Secondary antibodies were anti-rabbit IgG horseradish peroxidase linked (GE Healthcare NA9340V; 1: 10,000). ECL prime western blotting detection system (GE Healthcare) was used to develop the film (GE Healthcare).

Transmission Electron Microscopy

Cells adhered to coverslips were fixed in pre-warmed fixative (2.5% glutaraldehyde + 4% PFA (Agar Scientific) in 0.1 M PIPES (Sigma) buffer at pH 7.2) for 1 hour at room temperature, then incubated at 4 °C overnight. Cells were thoroughly washed in 0.1 M PIPES buffer 5 times for 15 minutes each. The fourth wash included 50 mM glycine in 0.1 M PIPES to quench free aldehydes. Cells then underwent a secondary fixation in 1% osmium tetroxide (TAAB Laboratories) in 0.1 M PIPES at 4 °C for 1 hour, after which they were washed in milliQ water 5 times for 10 minutes each. Cells then underwent a tertiary fixation in 0.5% uranyl acetate (Agar Scientific) overnight at 4 °C in the dark, then rinsed with milliQ water for 5-10 minutes. Cells were then dehydrated in 30%, 50%, 70%, 80%, 90% and 95% ethanol (Sigma), each for 10 minutes, then incubated in 100% ethanol for 90 minutes with 3 solution changes during this time. To infiltrate with epoxy resin, cells were incubated with 3:1 100% dry ethanol:Agar100 resin (Agar Scientific) for 1 hour, then 1:1 100% dry

ethanol:Agar100 resin for 2 hours, and 1:3 100% dry ethanol:Agar100 resin for 1 hour in a fume hood. Cells were then incubated in 100% Agar100 overnight at room temperature, and the resin changed twice the next day. The cells were embedded by inverting the coverslip onto Beem capsules filled with fresh 100% Agar100 resin. Blocks were polymerised for 24 hours at 60 °C, then submerged in liquid nitrogen and the coverslip snapped off, leaving cells embedded as a monolayer on the surface of the block. Ultrathin sections (90 nm) were taken using a Diatome diamond knife on the Leica UC7 ultramicrotome and mounted onto 200 mesh Cu grids. Sections were post-stained with Reynold's lead citrate (Reynolds, 1963) for 5 minutes, washed with degassed water and dried. Samples were transferred to a FEI Tecnai 12 transmission electron microscope and imaged at 120kV. Images were acquired using a Gatan OneView CMOS camera with Digital Micrograph 3.0 software.

Primary Antibodies

Antigen	Company	Dilution
**MBP**	Abcam	1:400
**NF200**	Sigma	1:400
	Abcam	1:10,000
**S100**	Dako	1:400
**BRN3A**	Millipore	1:500
**Collagen type-V**	Millipore	`1:500
**N-Cadherin**	BD Biosciences	1:400
**c-JUN**	Cell signalling	1:500
**SOX10**	Santa Cruz	1:500
**Krox20**	Covance	1:500
**Caspr**	Gift from Prof. Baht	1:400

**Pan-Na_V**	Sigma	1:400
**K_V1.2**	Neuromab	1:200
