# Supplementary figures and images for: Co-cultures with stem cell-derived human sensory neurons reveal regulators of peripheral myelination
Source: Brain. 2017 Feb 15;140(4):898–913. doi: 10.1093/brain/awx012 (PMC5637940; doi:10.1093/brain/awx012)

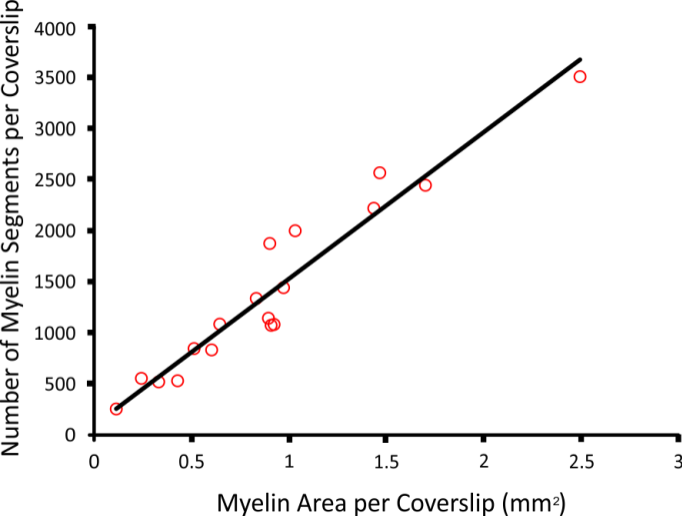

Supplement: Supplementary Data [file awx012_supp.zip › brain-2016-01328-File010.pdf]

NF200, MBP, DAPI

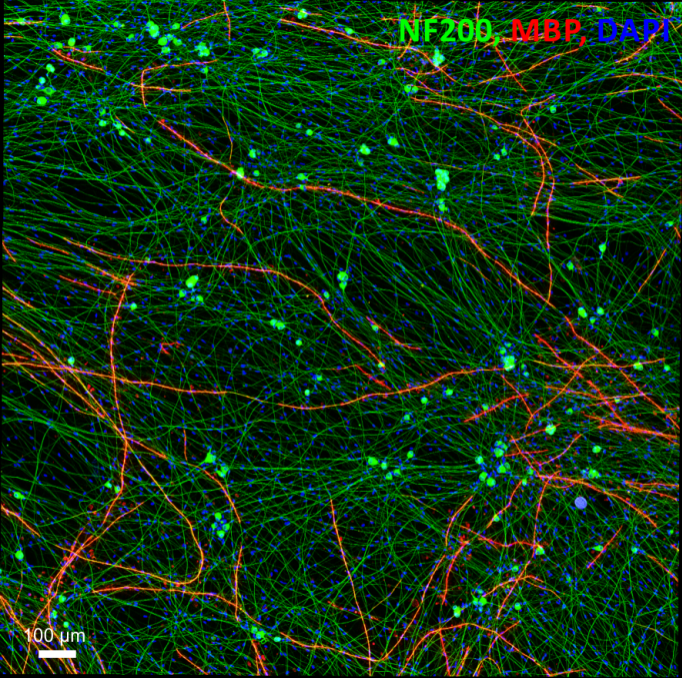

100 μm

Supplement: Supplementary Data [file awx012_supp.zip › brain-2016-01328-File011.pdf]

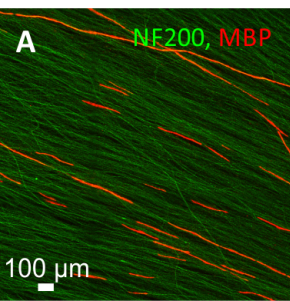

iPSC line 1

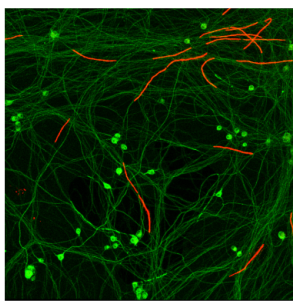

iPSC line 2

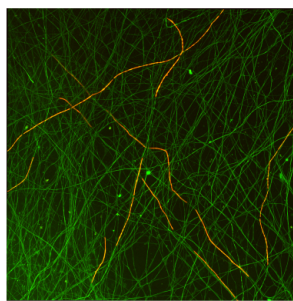

iPSC line 3

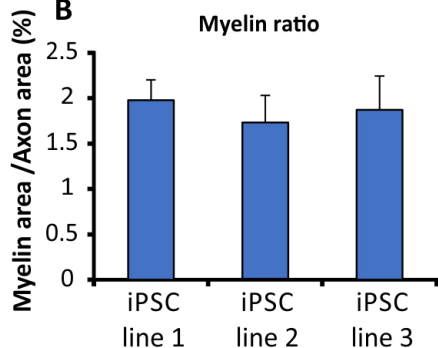

Supplement: Supplementary Data [file awx012_supp.zip › brain-2016-01328-File012.pdf]

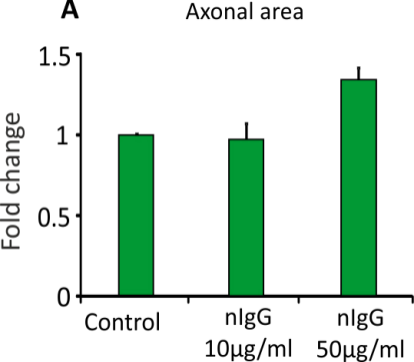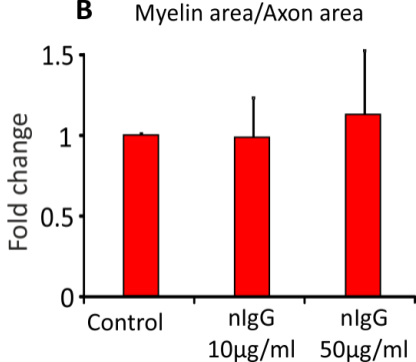

Supplement: Supplementary Data [file awx012_supp.zip › brain-2016-01328-File013.pdf]
